# Pregnancy Outcomes in a Cohort of Patients Who Underwent Double-J Ureteric Stenting—A Single Center Experience

**DOI:** 10.3390/medicina58050619

**Published:** 2022-04-29

**Authors:** Viorel Dragos Radu, Ingrid-Andrada Vasilache, Radu-Cristian Costache, Ioana-Sadiye Scripcariu, Dragos Nemescu, Alexandru Carauleanu, Valentin Nechifor, Veaceslav Groza, Pavel Onofrei, Lucian Boiculese, Demetra Socolov

**Affiliations:** 1 Urology Department, ‘Grigore T. Popa’ University of Medicine and Pharmacy, 700115 Iasi, Romania; vioreldradu@yahoo.com (V.D.R.); criscostache@hotmail.com (R.-C.C.); 2 Urology Department, ‘C.I. Parhon’ University Hospital, 700115 Iasi, Romania; grozaveaceslav@yahoo.com; 3 Department of Obstetrics and Gynecology, ‘Grigore T. Popa’ University of Medicine and Pharmacy, 700115 Iasi, Romania; isscripcariu@gmail.com (I.-S.S.); dnemescu@yahoo.com (D.N.); acarauleanu@yahoo.com (A.C.); valentinnechifor60@gmail.com (V.N.); demetrasocolov@gmail.com (D.S.); 4 Morphofunctional Sciences II Department, ‘Grigore T. Popa’ University of Medicine and Pharmacy, 700115 Iasi, Romania; onofrei.pavel@gmail.com; 5 Medical Informatics and Biostatistics Department, ‘Grigore T. Popa’ University of Medicine and Pharmacy, 700115 Iasi, Romania; lboiculese@gmail.com

**Keywords:** double-J stent, ureterohydronephrosis, urolithiasis, pyelonephritis, urosepsis, pregnancy

## Abstract

*Background and Objectives*: Minimally invasive procedures, such as double-J ureteric stenting, could be a promising therapeutic alternative to conservative management of obstructive urinary tract pathology. We aimed to evaluate the safety and effectiveness of double-J ureteric stenting in pregnant women with ureterohydronephrosis or urolithiasis, along with their infectious complications, and to assess the pregnancy outcomes of this cohort of patients in comparison with a control group. *Materials and Methods*: This observational retrospective study included 52 pregnant patients who underwent double-J ureteric stenting for urologic disorders in the Urology Department of ‘C.I. Parhon’ University Hospital, and who were followed up at a tertiary maternity hospital- ‘Cuza-Voda’, Iasi, Romania. The control group (63 patients) was randomly selected from the patient’s cohort who gave birth in the same time frame at the maternity hospital, without urinary pathology. Clinical, sonographic, and laboratory variables were examined. Descriptive statistics, non-parametric tests, and a one-to-one propensity score-matched analysis were used to analyze our data. *Results*: The univariate analysis indicated a significant statistical difference between the control group and the interventional group regarding maternal age (*p* = 0.018), previous maternal history of renal colic (*p* = 0.005) or nephrolithiasis (*p* = 0.002). After applying the propensity score-matched analysis, cesarean delivery rates (*p* < 0.001), preterm labour (*p* = 0.039), premature rupture of membranes (*p* = 0.026), preterm birth rates (*p* = 0.002), and post-partum UTI rates (*p* = 0.012) were significantly different between the control group and the matched treatment group. Ureterohydronephrosis, whether simple (*n* = 37; 71.2%) or infected (*n* = 13; 25%), was the main indication for double-J ureteric stenting. Complications such as pain (*n* = 21; 40.3%), stent migration (*n* = 3; 5.76%) or encrustation (*n* = 2; 3.84%), as well as reflux pyelonephritis (*n* = 2; 3.84%) and gross hematuria (*n* = 1; 1.92%) were recorded during follow-up. *Conclusions*: Our results show that double-J stenting is a safe and effective treatment option for pregnant patients with obstructive urological disorders.

## 1. Introduction

Ureterohydronephrosis (UHN) is a common maternal adaptation to pregnancy, affecting more than 40% of pregnancies, and is more prevalent in the third trimester [1]. The anatomical changes of the pyelocaliceal system are predominantly encountered in the right side, due to the anatomical relationship of the ureter with iliac and ovarian vessels [2]. Ureterohydronephrosis, along with hormonal and immune changes, predispose pregnant women to infectious complications that range between simple urinary tract infections to urosepsis [3]. 

Urolithiasis (UL) development during pregnancy is supported by systemic, nephrological and mechanical changes [4], and is associated with important adverse pregnancy outcomes such as preterm birth, preeclampsia or gestational hypertension [5]. If left untreated, obstructive uropathy can lead to urosepsis and renal failure, which could ultimately result in preterm birth, abruptio placentae, stillbirth, or maternal mortality [6,7,8].

Prenatal management of urinary tract pathology is based on correct diagnosis and individualized treatment. Ultrasonography (USG), magnetic resonance imaging (MRI), complete blood count (CBC), inflammatory markers (C-reactive protein—CRP), renal function tests, urinary analysis, and urine culture are useful diagnostic tools for detection of urinary tract disorders during pregnancy [5,9,10]. 

Individualized treatment of urinary pathology during pregnancy consists of a conservative (hydration, analgesia, and/or antibiotic treatment) and a surgical approach. The surgical approach is mainly represented by ureteric stent insertion, percutaneous nephrostomy (PCN) and ureteroscopic extraction of the calculus [11,12,13]. 

Ureteric stenting is a minimally invasive procedure with a good safety profile during pregnancy that can be used for drainage of the obstructed and/or infected urinary system in patients with symptoms refractory to conservative approaches and/or changes in renal function, pain visual analogue score, obstruction or hydronephrosis grading [14]. The procedure can be easily performed in the lithotomy position, without general anaesthesia [15]. 

The aim of this study was to evaluate the safety and effectiveness of double-J ureteric stenting in pregnant women with ureterohydronephrosis and renal obstruction due to calculi or physiological obstruction, along with their infectious complications, and to assess the pregnancy outcomes of this cohort of patients in comparison with a control group. 

## 2. Materials and Methods

We conducted an observational retrospective study of all pregnant patients who underwent double-J ureteric stenting for urologic disorders (ureterohydronephrosis, urolithiasis, and their infectious complications) in the Urology Department of ‘C.I. Parhon’ University Hospital, Iasi, Romania, between January 2014 and December 2020. All patients were followed up at a tertiary maternity hospital- ‘Cuza-Voda’, Iasi, Romania. The control group was randomly selected from the patient’s cohort who gave birth in the same time frame at the maternity hospital, without urinary tract pathology.

Ethical approval for this study was obtained from the Institutional Ethics Committees of ‘Cuza-Voda’ Maternity Hospital (No. 2871/05.03.2022) and ‘C.I. Parhon’ University Hospital (No. 1808/04.03.2022). Informed consent was obtained from all participants included in the study. All methods were carried out in accordance with relevant guidelines and regulations. 

Medical records of patients were systematically reviewed and data obtained. Exclusion criteria comprised patients who had multiple pregnancies, ectopic pregnancies, first and second trimester abortions, fetal intrauterine demise, fetuses with chromosomal or structural abnormalities, intrauterine infection, incomplete medical records, incorrect/lack of first trimester sonographic pregnancy dating or who were unable to offer informed consent due to various reasons (age less than 18 years old, intellectual deficits, psychiatric disorders, etc.). 

A total of 284 pregnant women with urological disorders were admitted at ‘C.I. Parhon’ University Hospital during our study period. Pregnant patients who underwent double-J ureteric stenting were evaluated, and 52 patients were included in our study. The following variables were recorded: demographic data, the patient’s medical history, renal clinical manifestations (febrile syndrome and renal colic) laboratory parameters (CBC, CRP, urinalysis and urine culture), indications for double-J ureteric stenting, duration of the procedure and hospitalization, associated medical treatment, type of complications, and pregnancy outcomes (type of birth, newborn’s gender, Apgar score, preterm labour, premature rupture of membranes, preterm birth, fetal growth restriction, preeclampsia, neonatal intensive care unit (NICU) admission, fetal death, and post-partum UTI). The Apgar score, developed by Dr. Virginia Apgar, was used to assess the status of infants after delivery [16]. It comprises 5 components: (1) color; (2) heart rate; (3) reflexes; (4) muscle tone; and (5) respiration [17]. Each of these components is given a score of 0, 1, or 2. An Apgar score less than 7 indicated the need for special neonatal care, while a score between 7 and 10 was considered reassuring. 

All pregnancies were dated by an experienced obstetrician with an early ultrasound scan using an E8/E10 (General Electric Healthcare, Zipf, Austria) scanner with a 4.8 MHz transabdominal probe (GE Medical Systems, Milwaukee, WI) between 10 + 0 to 13 + 6 weeks to determine gestational age by measuring the crown-rump length (CRL) [18].

Ultrasound evaluation was also performed by experienced urologists for diagnostic purposes using Siemens ACUSON ×300 or ACUSON REDWOOD (Siemens Healthcare, Erlangen, Germany, gmbH) scanners, with a 3.5 MHz transabdominal probe. At ultrasound evaluation, we could only assess calculi in the pyelocaliceal system, lumbar and pelvic ureter. The patients presenting with UHN without an ultrasound objectification of a calculi were considered as no-lithiasis patients. 

UHN grading, adapted after the Society for Fetal Urology (SFU) system, was considered as follows: (a) grade I: minimal changes in urinary stasis; (b) grade II: slight dilation of the renal pelvis involving major calyces; (c) grade III: moderate dilation of the renal pelvis involving major and minor calyces; (d) stage IV: severe dilation with compression on the renal parenchyma [19,20]. 

In the presence or absence of cystitis symptoms, flank pain, nausea/vomiting, temperature (>38 °C), and/or costovertebral angle tenderness were used as diagnostic criteria for acute pyelonephritis [21] More than 10^5^ colony-forming units (CFU)/mL was considered a positive urine culture.

Urosepsis was diagnosed in patients meeting two or more of the following criteria according to the quick sepsis-related organ failure assessment (qSOFA): (1) respiratory rate of ≥22 breaths/min; (2) altered consciousness (Glasgow Coma Scale score of <13); (3) systolic blood pressure of ≤100 mmHg [22].

The ureter was stented under local anaesthesia, using pregnancy-approved antibiotic prophylaxis. The procedure was conducted using an Olympus rigid cystoscope, 21CH. A 6, 7 or 8CH, 26, 28 cm length, JJ ureteric stent (MEDpro Medical B.V., Fernendal, The Netherlands) was inserted retrogradely over a guidewire. The ureteric stent’s location was confirmed by observing the stent markings and distal coiling, as well as intraoperative sonographic stent placement inside the pyelocaliceal system. During the procedure, no fluoroscopy was employed.

When the symptomatology subsided or the urosepsis cleared, the patients were discharged. All recommended antibiotic regimens followed the European Association of Urology guidelines [23]. 

Statistical analysis was performed using SPSS software (version 28.0.1, IBM Corp, Armonk, NY, USA). Each variable was evaluated with chi-squared and Fisher’s exact tests for categorical variables, and T-tests for continuous variables. One-to-one propensity score-matched analysis was performed using Stata SE (version 15, StataCorp LLC), considering demographic characteristics as treatment independent variables, and comparing pregnancy outcomes (type of birth, preterm labour, premature rupture of membranes, preterm birth, neonatal intensive care unit admission rates, and post-partum UTI rates) between the control group (without double-J stent) and treatment group (with double-J stent). A *p* value less than 0.05 was considered statistically significant. 

## 3. Results

A total of 52 pregnant patients who underwent double-J ureteric stenting were included in our study. A group of 63 patients, who gave birth at ‘Cuza Voda’ Hospital, without urological illnesses and interventions during pregnancy served as our control group. The demographic characteristics, comorbidities, and pregnancy outcomes of cases and controls are presented in Table 1.

Our data indicated a significant statistical difference between the control group and the interventional group regarding maternal age (*p* = 0.018), previous maternal history of renal colic (*p* = 0.005) or nephrolithiasis (*p* = 0.002), type of birth (*p* < 0.001), newborn’s Apgar score at 5 min (*p* = 0.007), preterm labour rates (*p* = 0.03), premature rupture of membranes (*p* = 0.006), preterm birth (*p* = 0.002), neonatal intensive care unit (NICU) admission rates (*p* = 0.03), and post-partum UTI rates (*p* < 0.001). 

After applying the one-to-one propensity score-matched analysis, only cesarean delivery rates (*p* < 0.001), preterm labour (*p* = 0.039), premature rupture of membranes (*p* = 0.026), preterm birth rates (*p* = 0.002), and post-partum UTI rates (*p* = 0.012) were significantly different between the control group and the matched treatment group (Table 2). 

The most frequent pathogens responsible for post-partum UTI were *Escherichia coli* (interventional vs. control groups, 3:1 cases), *Klebsiella* spp. (interventional vs. control groups, 3:1 cases), *Enterococcus* spp. (interventional vs. control groups, 3:0 cases), followed by *Enterobacter* spp. (interventional vs. control groups, 1:0 cases) and *Staphylococcus* spp. (interventional vs. control groups, 1:0 cases). These UTI were treated with Amoxicillin-clavulanic acid 1 g b.i.d or Cefuroxime 1.5 g b.i.d in the post-partum period for 10–14 days.

A total of 52 pregnant patients underwent double-J ureteric stenting in the Urology Department. The mean gestational age at the moment of the procedure was 23.21 ± 7.11 weeks (Table 3). The majority of the procedures were performed in the second trimester (*n* = 29, 55.8%), and only five procedures were performed in the first trimester of pregnancy. 

The mean values and standard deviations (SDs) of pre-procedural leukocytosis and serum CRP were 15,345.23 ± 2340.4/mm^3^ and 123.32 ± 46 mg/L, respectively. We observed a significant reduction in the post-procedural leukocytosis (8650.8 ± 1890.3/mm^3^), but we did not evaluate the post-procedural CRP levels due to a slower resolution of the inflammatory syndrome. 

The main pathogens that determined urinary tract infection were *Escherichia coli* (*n* = 19; 36.5%), *Klebsiella* spp. (*n* = 5; 9.61%), *Enterococcus* spp. (*n* = 4; 7.6%), followed by *Serratia* spp. (*n* = 2; 3.8%) and *Staphylococcus* spp. (*n* = 1; 1.92%). Only 4 cases (7.6%) had UTIs resulting from multidrug resistant *Escherichia coli*, and all of them developed a septic condition.

Antibioprophilaxis, consisting of Ceftriaxone 2 g b.i.d, was administered before procedure, and was continued for 14 days, if the urinary infection was confirmed by a positive culture of urine. Pyelonephritis in pregnancy was treated with either intravenous Amoxicillin Amoxicillin 1 g q.d.s plus Gentamicin 5 mg/kg/day for 14 days or Ceftriaxone 2 g b.i.d. Urosepsis cases were evaluated in the intensive care unit, and received Ceftriaxone 2 g b.i.d, Piperacillin/tazobactam 4.5 g t.i.d or Meropenem 1 g t.i.d (for multiresistant bacteria), along with supportive treatment. 

Ureterohydronephrosis, whether simple (*n* = 37; 71.2%) or infected (*n* = 13; 25%), was the main indication for double-J ureteric stenting in our cohort of patients. The right side was the most affected, and bilateral occurrence of the simple UHN was encountered in three cases (5.8%), while bilateral infected UHN manifested in two cases. (3.8%)

The mean duration of the procedure was 28.32 ± 13.4 min. Double-J ureteric stent insertion was performed in 46 cases (88.4%), while in six cases (11.6%) double-J stents were replaced because they were in place for at least 6 weeks. We used only local anesthesia for performing these procedures. The mean duration of the hospitalization in the Urology Department was 5.19 ± 1.98 days.

In two cases, additional procedures were required. In one case, retrograde ureteroscopy with lithotripsy (*n* = 1; 1.92%) was performed for a pelvic ureteral calculus, followed by double-J stent insertion. Because a wire could not be passed through the cystoscope and the obstructive calculus was smaller than 10 mm in diameter, we performed retrograde ureteroscopy with lithotripsy. In another case, due to significant calcification of the distal end of the double- stent, endoscopic lithotripsy (*n* = 1; 1.92%) was performed in order to retrieve the stent for replacement. Intravenous general anesthesia without oro-tracheal intubation was performed in these two cases.

A total of 21 patients (40.3%) reported pain or urinary discomfort after the procedure, and we administered intravenous Acetaminophen 1 g b.i.d, Metamizole 1 g b.i.d or Drotaverine hydrochloride 40 mg b.i.d. Stent migration (*n* = 3; 5.76%) or encrustation (*n* = 2; 3.84%), as well as reflux pyelonephritis (*n* = 2; 3.84%) and gross hematuria (*n* = 1; 1.92%) were other complications recorded during follow-up. No maternal or fetal death was recorded following the procedure. 

## 4. Discussion

In this retrospective study, we assessed the safety and effectiveness of double-J ureteric stenting in pregnant women with ureterohydronephrosis, renal obstruction due to calculi or physiological ureteral obstruction, and their infectious complications, as well as the pregnancy outcomes of this cohort of patients in comparison with a control group.

Our results showed that the double-J stenting is a safe and effective treatment option for pregnant patients. Most of the procedures were performed during the second trimester. It was reported that double-J ureteric stenting can be difficult in the third trimester due to the tortuosity of the ureter [24]. However, we did not confirm this aspect because all our patients had successful stenting, regardless of the trimester of pregnancy. 

The main indication for these procedures was UHN, simple (71.2%) or complicated with infection (25%). Right ureterohydronephrosis was the most frequently encountered in our cohort of patients. This aspect can be explained by the dextrorotation of the uterus during pregnancy with subsequent vascular compression, and by the anatomical relationship of the ureter with iliac and ovarian vessels [25,26]. All patients had complete resolution of the hydronephrosis on follow-up renal ultrasound and regression of hydronephrosis and urinary symptoms after ureteral stenting.

*Escherichia coli*, *Klebsiella* spp., and *Enterococcus* spp. were the main determinants of infectious complications in our cohort of patients. This bacterial spectrum corresponds to the pathogenic microorganisms associated with UTI in pregnancy [27,28,29]. All patients underwent antibioprophilaxis with a third-generation cephalosporin, Ceftriaxone, which was continued for 14 days if an UTI was confirmed by urine culture. 

Complicated UTIs such as pyelonephritis were treated with either a combination of intravenous Amoxicillin plus Gentamicin or Ceftriaxone for two weeks. The use of an aminoglycoside was considered after weighting the risks and benefits of this drug during pregnancy, and all patients agreed with its administration after a proper counseling. Current literature data, although limited, does not support an association between Gentamicin use and increased risk of birth defects or audiologic deficits [30,31,32]. 

In the case of urosepsis, supplementary antibiotic options included Piperacillin/tazobactam and Meropenem along with supportive treatment, as recommended by current guidelines [23,33]. Meropenem was reserved for the treatment of severe infections caused by extended-spectrum β-lactamases (ESBL) positive *Escherichia coli*.

The mean duration of the procedure was 28.32 ± 13.4 min. Unilateral placement of the double-J ureteric stent was performed in the majority of cases (*n* = 46; 88.4%), and in 12 cases (23%) double-J stents were replaced because they were in place for at least 6 weeks. The mean duration of the hospitalization was 5.19 ± 1.98 days. We must emphasize that all procedures of double-J insertion were made in an emergency regime due to the known fact that lumbar pain secondary to physiologic ureteral obstruction, symptomatic lithiasis, and ascending urinary infections carry a higher risk for poor maternal and fetal outcomes [34,35,36]. We preferred to rapidly solve the urinary stasis and avoid the prolonged conservative approach reported in other studies [37,38,39]. Furthermore, we postponed ureteroscopy or percutaneous nephrolithotomy until after birth. 

In two cases, additional procedures were required. Retrograde ureteroscopy with lithotripsy was performed for a pelvic ureteral calculus, followed by double-J stent insertion. Due to significant calcification of the distal end of the double-J stent, endoscopic lithotripsy was performed for another case in order to retrieve the stent for replacement. In our study, we replaced 12 double-J stents that were placed for more than 6 weeks. It has been reported that if a double-J stent is indwelled for 6 weeks, 6–12 weeks, or greater than 12 weeks, the rates of stent encrustation are 9.2 percent, 47.5 percent, and 76.3 percent, respectively [40]. The general opinion is that within 6 weeks to 6 months, the stent should be replaced or removed [40,41,42]. After birth, the stents were removed, and we performed a urography to identify calculi.

In our cohort of patients, the complication rates were low, and no fetal or maternal morbidity was recorded. Similar rates of stent migration, stent encrustation, reflux pyelonephritis, and hematuria were reported in other series of pregnant patients [15,43]. 

As for pregnancy outcomes, it appeared that the cesarean rate was significantly higher in the interventional group compared to the control group (34.8% vs. 24.3%), and the Apgar score at birth appeared to be significantly lower in the interventional group (8.02 ± 1.56 vs. 8.73 ± 1.24). 

Preterm labour, as well as premature rupture of membrane rates, were significantly higher in the interventional group. Tocolysis included calcium channel blockers (Nifedipine), magnesium sulphate (also used for neuroprotection) or betamimetics (Hexoprenaline). The results from the univariate analysis showed that preterm birth rates, as well as NICU admission rates, were significantly higher in the interventional group. However, the propensity score match analysis did not support the higher NICU admission rates in the control group. No neonatal death was recorded. Similar findings were outlined in pregnant patients with a urologic pathology [44,45].

Postpartum UTIs were more frequently encountered in the interventional group, and the bacterial spectrum responsible for postpartum UTIs was similar to that described in the pre-procedural urine culture, with *Escherichia coli*, *Klebsiella* spp., and *Enterococcus* spp. being the most prevalent. The higher risk of postpartum urinary infection can be explained by the presence of double-J stents, which increase this risk, and by low patient compliance, persistent infections, or reinfections. 

The main limitation of our study was that we could not include the majority of pregnant patients operated on at ‘Parhon Hospital’ during the selected period due to the lack of data about obstetrical outcomes. Other limitations included the retrospective and unicentric study design. A greater cohort of patients recruited from multiple centers would allow a more comprehensive picture of the issue.

## 5. Conclusions

In this study, we provided consistent data that support our approach of urologic emergencies during pregnancy. Double-J stenting is a safe and effective procedure that can be easily performed throughout pregnancy using only local anesthesia.

Further research is needed to evaluate larger cohorts of patients with various poor obstetrical outcomes possibly associated with the double-J ureteric stenting procedure.

## Figures and Tables

**Table 1:** Demographic characteristics, comorbidities, and pregnancy outcomes of the evaluated groups.

Variable		Without Double-J Stent (63 Patients)	With Double-J Stent (52 Patients)	*p* Value
Demographic characteristics	Age	28.7 ± 5.75	26.12 ± 5.74	0.018
	Number of gestations	2.25 ± 1.45	2.40 ± 2.13	0.65
	Parity	1.87 ± 1.28	2.02 ± 1.87	0.62
Comorbidities	Previous cesarean section	No 53 (46.1%) Yes 10 (8.7%)	No 44 (38.3%) Yes 8 (7%)	0.94
	Placenta praevia	No 58 (50.4%) Yes 5 (4.3%)	No 48 (41.7%) Yes 4 (3.5%)	0.96
	Gestational hypertension	No 57 (49.6%) Yes 6 (5.2%)	No 50 (43.5%) Yes 2 (1.7%)	0.23
	Preeclampsia	No 62 (53.9%) Yes 1 (0.8%)	No 49 (42.6%) Yes 3 (2.6%)	0.22
	Previous renal colic	No 61 (53%) Yes 2 (1.7%)	No 42 (36.5%) Yes 10 (8.6%)	0.005
	Previous nephrolithiasis	No 62 (53.9%) Yes 1 (0.8%)	No 43 (37.3%) Yes 9 (7.8%)	0.002
	In vitro fertilization	No 62 (53.9%) Yes 1 (0.9%)	No 51 (44.3%) Yes 1 (0.9%)	0.89
Pregnancy outcomes	Type of birth	Cesarean 28 (24.3%) Vaginal 35 (30.4%)	Cesarean 40 (34.8%) Vaginal 12 (10.4%)	<0.001
	Newborn’s gender	Female 31 (27%) Male 32 (27.8%)	Female 26 (22.6%) Male 26 (22.6%)	0.93
	Apgar score	8.73 ± 1.24	8.02 ± 1.56	0.007
	Preterm labour	No 54 (46.9%) Yes 9 (7.8%)	No 36 (31.3%) Yes 16 (13.9%)	0.03
	Premature rupture of membranes	No 62 (53%) Yes 1 (1.7%)	No 44 (33%) Yes 8 (12.1%)	0.006
	Preterm birth	No 61 (53%) Yes 2 (1.7%)	No 38 (33%) Yes 14 (12.1%)	0.002
	Fetal growth restriction	No 61 (53%) Yes 2 (1.7%)	No 47 (40.8%) Yes 5 (4.3%)	0.15
	Preeclampsia	No 62 (53.9%) Yes 1 (0.9%)	No 51 (44.3%) Yes 1 (0.9%)	0.89
	NICU admission	No 59 (51.3%) Yes 4 (3.4%)	No 42 (36.5%) Yes 10 (8.6%)	0.03
	Fetal death	0 (0%)		
	Post-partum UTI	No 61 (53%) Yes 2 (1.7%)	No 40 (34.8%) Yes 12 (10.4%)	<0.001

**Table 2:** Results from the propensity score match analysis.

Outcome	Robust Standard Error (RSE)	Coefficient	95% Confidence Interval		*p* Value
			Lower Limit	Upper Limit	
Type of birth (cesarean)	0.089	0.362	0.186	0.538	<0.001
Preterm labour	0.091	0.160	−0.018	0.340	0.039
Premature rupture of membranes	0.055	0.105	−0.002	0.214	0.026
Preterm birth	0.075	0.236	0.087	0.384	0.002
Fetal growth restriction	0.050	0.056	−0.041	0.15	0.26
Preeclampsia	0.018	−0.008	−0.044	0.027	0.63
NICU admission	0.064	0.086	−0.039	0.213	0.17
Post-partum UTI	0.066	0.166	0.036	0.296	0.012

**Table 3:** Summary of double-J ureteric stenting.

Variable	Mean (±SD) or *n* (%)
Timing of the procedure (weeks of gestation/trimester of pregnancy)	Mean (SD) 23.21 ± 7.11 First trimester 5 (9.6%) Second trimester 29 (55.8%) Third trimester 18 (34.6%)
Leukocytosis	Pre-procedure 15,345.23 ± 2340.4/mm^3^ Post-procedure 8650.8 ± 1890.3/mm^3^
CRP	Pre-procedure 123.32 ± 46 mg/l
Urinalysis and urine culture	Leukocyturia 31 (59.6%) *Escherichia coli* 19 (36.5%) *Klebsiella* spp. 5 (9.61%) *Enterococcus* spp. 4 (7.6%) *Serratia* spp. 2 (3.8%) *Staphylococcus* spp. 1 (1.92%)
Indications for double-J stenting	Simple UHN 37 (71.2%) Simple UHN location: Left 11 (21.2%) Right 23 (44.2%) Bilateral 3 (5.8%) Infected UHN 13 (25%) Infected UHN location: Left 1 (1.9%) Right 10 (19.2%) Bilateral 2 (3.8%) UHN grade 1.67 ± 0.51 Urolithiasis 14 (26.9%) Urolithiasis location: Left 5 (9.6%) Right 8 (15.4%) Bilateral 1 (1.9%) Urosepsis 15 (28.8%) Pyelonephritis 3 (5.7%) Pyelonephritis location: Left 1 (1.9%) Right 2 (3.8%)
Types of interventions	Double-J ureteric stent insertion *n* = 46 (88.4%) Double-J ureteric stent replacement *n* = 6 (11.6%)
Associated procedures	Retrograde ureteroscopy with lithotripsy 1 (1.92%) Endoscopic lithotripsy 1 (1.92%)
Complications	Pain/ urinary discomfort 21 (40.3%) Stent migration 3 (5.76%) Stent encrustation 2 (3.84%) Reflux pyelonephritis 2 (3.84%) Gross hematuria 1 (1.92%)
Duration of hospitalization	5.19 ± 1.98 days
Duration of the procedure	28.32 ± 13.4 min

## Data Availability

The data presented in this study are available on request from the corresponding author. The data are not publicly available due to local policies.
